# Domestic Asbestos Exposure: A Review of Epidemiologic and Exposure Data

**DOI:** 10.3390/ijerph10115629

**Published:** 2013-10-31

**Authors:** Emily Goswami, Valerie Craven, David L. Dahlstrom, Dominik Alexander, Fionna Mowat

**Affiliations:** 1 Exponent, 475 14th Street, Suite 400, Oakland, CA 94612, USA; E-Mail: cravenv@exponent.com; 2 New Era Sciences, LLC, Issaquah, WA 98027, USA; E-Mail: dldahlstrom@nes2013.com; 3 Exponent, 2595 Canyon Boulevard, Suite 440, Boulder, CO 80303, USA; E-Mail: dalexander@exponent.com; 4 Exponent, 149 Commonwealth Drive, Menlo Park, CA 94025, USA; E-Mail: fmowat@exponent.com

**Keywords:** domestic, exposure, epidemiology, asbestos fibers, take-home

## Abstract

Inhalation of asbestos resulting from living with and handling the clothing of workers directly exposed to asbestos has been established as a possible contributor to disease. This review evaluates epidemiologic studies of asbestos-related disease or conditions (mesothelioma, lung cancer, and pleural and interstitial abnormalities) among domestically exposed individuals and exposure studies that provide either direct exposure measurements or surrogate measures of asbestos exposure. A meta-analysis of studies providing relative risk estimates (n = 12) of mesothelioma was performed, resulting in a summary relative risk estimate (SRRE) of 5.02 (95% confidence interval [CI]: 2.48–10.13). This SRRE pertains to persons domestically exposed via workers involved in occupations with a traditionally high risk of disease from exposure to asbestos (*i.e.*, asbestos product manufacturing workers, insulators, shipyard workers, and asbestos miners). The epidemiologic studies also show an elevated risk of interstitial, but more likely pleural, abnormalities (n = 6), though only half accounted for confounding exposures. The studies are limited with regard to lung cancer (n = 2). Several exposure-related studies describe results from airborne samples collected within the home (n = 3), during laundering of contaminated clothing (n = 1) or in controlled exposure simulations (n = 5) of domestic exposures, the latter of which were generally associated with low-level chrysotile-exposed workers. Lung burden studies (n = 6) were also evaluated as a surrogate of exposure. In general, available results for domestic exposures are lower than the workers’ exposures. Recent simulations of low-level chrysotile-exposed workers indicate asbestos levels commensurate with background concentrations in those exposed domestically.

## 1. Introduction

The potential exposure scenarios for individuals who are non-occupationally exposed to asbestos vary, but may include: (1) neighborhood exposure due to asbestos emissions from nearby asbestos-product manufacturing facilities, asbestos mines, construction work involving asbestos, or naturally occurring asbestos; (2) household exposure from the use of asbestos-containing materials (e.g., use of tremolite/erionite whitewash on the exterior of homes); and (3) household contamination resulting from asbestos fibers brought into the home on workers’ clothing or bodies, and domestic activities such as handling or laundering workers’ contaminated clothing. In this review, we discuss the third scenario, which can be referred to as secondary, para-occupational or take-home exposure, and herein is termed “domestic exposure”.

### Early Reports of Domestic Exposure

In 1960, a seminal case series reported by Wagner [1] was not only one of the first to associate asbestos exposure, specifically to crocidolite, with the development of malignant pleural mesothelioma in 33 persons, but was also the first to identify exposure pathways via non-occupational domestic and neighborhood asbestos exposure. Wagner’s study was followed shortly by a case-control study by Newhouse and Thompson [2,3], which identified seven cases of pleural mesothelioma and two cases of peritoneal mesothelioma in patients whose relatives worked with asbestos, including chrysotile, amosite, and crocidolite. These workers’ occupations included spinners, an engine-room worker, a boiler coverer, an asbestos factory foreman, a docker handling asbestos cargo, a railway carriage builder, and an asbestos factory worker. Later studies involving domestically exposed persons followed (e.g., [4,5]).

The review of indirect exposures in bystanders in the workplace and at home by Grandjean and Bach [6] provided a relatively early evaluation of indirect exposures to lead, beryllium, asbestos, and other substances, including bystander exposures and exposure to substances carried home from work by family members. The authors addressed early case reports, case series, and cross-sectional studies that documented cases of mesothelioma, lung cancer, asbestosis, and pleural plaques in persons believed to be exposed domestically through family members who worked mostly in shipyards or asbestos factories. No specific data on the number of persons included in their evaluation were provided, nor was information on fiber type provided. 

In conjunction with the Workers’ Family Protection Act of 1992, the U.S. National Institute for Occupational Safety and Health (NIOSH) produced the Report to Congress on Workers’ Home Contamination Study Conducted under the Workers’ Family Protection Act [7]. The authors of this report evaluated “the potential for, prevalence of, and issues related to the contamination of workers’ homes with hazardous chemicals and substances...transported from the workplaces of such workers”. In their report, NIOSH indicated that they included four cohort studies, one community study, seven case-control studies, and “numerous” case reports and case series. This report is the most comprehensive by NIOSH to date on this topic—it provides a summary of cohort studies, case-control studies, case reports, and case series, as well as an overview of studies that describe contaminated clothing. The report concluded that domestic asbestos exposures may pose an increased risk of disease, but did not provide analyses regarding the type of exposure (including fiber type), level, frequency, or duration needed to produce disease. As a follow-up to this report, NIOSH published a research agenda focused on protecting workers’ families [8]. This agenda included characterizing the extent of home contamination, identifying populations at greatest risk of known and suspected take-home exposures, assessing the adverse health effects from take-home exposures, and assessing the effectiveness of prevention and remediation methods. To date, NIOSH has not published any results from this agenda. 

An often-cited French report by the National Institute of Health and Medical Research (INSERM) [9] concluded that the risk of mesothelioma in persons exposed in a non-occupational and domestic setting was “established” in the literature, indicating that the source of the asbestos was typically dirty work clothes; however, this report does not provide any quantitative domestic exposure estimates, and it specifically states that good exposure data do not exist in the literature to feasibly evaluate the extent of domestic exposures. In addition, the authors lump non-occupational (termed “para-occupational” by the original authors) exposures together, which include domestic and direct exposures from the use of home products potentially containing asbestos (e.g., ironing boards and insulating gloves), thereby making it difficult to understand those exposures that resulted solely from domestic exposure. No specific data on the number of persons included in their evaluation were provided.

In 2000, Bourdès *et al.* [10] conducted a study focused solely on pleural mesothelioma based on five published studies, and the reported meta-relative risk included a study of household use of asbestos (e.g., whitewash, stucco) in Turkey in which 23 mesotheliomas were reported; however, a number of studies have been published since its culmination date of 1998. Three of the remaining four included studies provided information on the number of domestically-exposed persons with mesothelioma, reporting on a combined 21 cases of mesothelioma; the fifth included study did not provide information by exposure type, but noted that 17 persons (9%) likely or possibly had para-occupational exposure. In all but one of the included studies, the exposure was to amosite or mixed fibers (*i.e.*, amphibole and chrysotile fibers). Bourdès *et al.* also presented a review of non-occupational exposure measurements, which largely included environmental exposures (ambient exposures due to nearby sources) and indoor exposures due to specific asbestos-containing products used in the home or business (e.g., schools with sprayed asbestos, use of asbestos-containing whitewash in the home). 

The purpose of our current paper has two specific aims: (1) to provide an up-to-date and comprehensive review of epidemiologic (cohort, case-control, and case reports and series) and exposure data regarding domestic exposure and mesothelioma, lung cancer, and interstitial and pleural abnormalities and (2) to conduct a quantitative assessment using a meta-analysis approach to estimate the risk of mesothelioma among individuals domestically exposed *vs.* those not exposed. The issue of domestic exposures remains an important question because of potential ongoing uses of potentially hazardous materials. For asbestos, the issue becomes important because of ongoing litigation matters and the need to understand historical exposures to asbestos and the associations with asbestos-related diseases. During the time of writing this paper, another paper has been published which also provides a review of epidemiologic and exposure data regarding domestic exposures [11]; however, this paper does not provide a meta-analysis or quantitative evaluation of risk, excludes several studies that are included in the present paper, and uses different methods of evaluation.

## 2. Methods

The published literature from the 1960s to 2012 was searched using MEDLINE, accessed via PubMed (the U.S. National Library of Medicine). Key words included domestic, household, laundry, para-occupational, or take-home and asbestos (and specific fiber types, including crocidolite, amosite, and chrysotile), mesothelioma, lung cancer, asbestosis, or pleural changes. No specific restrictions were imposed on the literature search, although the review was restricted to the most recent update of a study population. The reference lists of articles were reviewed to identify studies that might not have been detected in the literature search. Each article was reviewed by at least two scientists for inclusion. In an attempt to be as comprehensive as possible, all studies that provided some primary epidemiologic or exposure information were included. Some studies were written in a foreign language; for these, the English abstract was relied upon for relevant information.

### 2.1. Epidemiology Review and Analysis

Analytical and descriptive epidemiologic studies were considered in the qualitative review, including cohort, case-control, cross-sectional studies, case reports, and case series. The medical conditions of interest were mesothelioma, lung cancer, and interstitial and pleural abnormalities. 

In addition to a qualitative review of the published epidemiologic studies, we also performed a quantitative meta-analysis of the studies reporting mesothelioma in domestically exposed persons. Only mesothelioma studies were included in the meta-analysis, because there were too few studies of lung cancer and interstitial and pleural abnormalities to perform meta-analyses for those endpoints. Epidemiologic studies were included in the meta-analysis if the original study reported relative risk estimates, or provided the information necessary to calculate a relative risk estimate, and a measure of variance (e.g., confidence intervals). Random-effects meta-analysis models were used to calculate summary relative risk estimates (SRREs), 95% confidence intervals (CIs), and corresponding *p*-values for heterogeneity (p-H). Statistical significance was identified when the 95% CI did not include 1.0. The random-effects model assumes that the study-specific effect sizes come from a random distribution of effect sizes according to a specific mean and variance. A p-H < 0.1 suggests significant “between-study” statistical variability in a meta-analysis model [12]. The relative risk estimates of the individual studies were weighted based on the inverse of the variance, which is related to the sizes of the study populations. Tests for heterogeneity were conducted, and subgroup analyses (specifically, case-control *vs.* cohort, modification by occupational exposure) were performed to discern any potential sources of between-study variability. “One study removed” sensitivity analyses were conducted to evaluate the relative influence of each study on the model-specific SRRE. This was performed by generating an SRRE based on all studies in a particular model, followed by the removal of one study at a time to compare the overall SRRE with SRREs from models that had one study removed. Separate models were created to estimate the effects of occupational *vs.* neighborhood exposures. Potential confounding from occupational or neighborhood exposures was assessed by the methods described in each paper, as well as suggestions from the original authors’ discussion of limitations. Analyses were conducted using Comprehensive Meta-Analysis (version 2.2.045; Biostat, Englewood, NJ, USA), STATA (version 1.0; StataCorp, College Station, TX, USA), and Episheet [13].

### 2.2. Exposure Review

The exposure studies reviewed included a variety of study types that provided some direct asbestos exposure data or surrogate of asbestos exposure, and were categorized into four distinct groups: (1) studies describing results of airborne or settled dust samples collected within the homes of domestically exposed persons, (2) studies describing exposures during laundering or other handling of contaminated clothing, (3) studies describing controlled simulations of take-home exposures, and (4) lung burden studies. Due to the different in potency among asbestos fiber types, wherever possible, the type of asbestos fiber from which the exposure occurred is noted.

## 3. Results

In total 143 published articles were identified for inclusion in the review, and of these, 108 were evaluated for relevant information. Many of the studies were subsequently excluded after initial review. Specific reasons for exclusion included lack of quantitative data regarding risk and/or exposure (e.g., review articles with no original data) and studies that did not report specifically on domestically-exposed persons or that lumped those domestically-exposed with other types of asbestos exposures. The remaining articles are discussed below. Wherever possible, the asbestos fiber type to which the population was exposed was reported (Table 1, Table 2 and Table 3).

### 3.1. Review of Domestic Epidemiologic Studies

Studies of mesothelioma, lung cancer, and pleural and interstitial abnormalities with information regarding domestic exposure are discussed below by disease type. 

#### 3.1.1. Mesothelioma

Table 1 provides a list of 32 case reports and case series of mesothelioma in asbestos-exposed domestic populations, beginning with Wagner and colleagues’ case series of pleural mesothelioma in 1960. The case reports and series are provided for comprehensiveness, not to address the question of whether or not there is an association between domestic exposure to asbestos and mesothelioma, or the magnitude of that association. 

**Table 1:** Case reports and series of mesothelioma in domestically exposed populations.

Author and Year	Population Studied	Occupation of Worker(s)	Results	Exposure Information
Wagner *et al.* 1960 [1]	33 Pleural mesothelioma cases in Northwest Cape Province (South Africa)	Crocidolite miners	25/33 cases had non-occupational exposure (76%).	Nearly exclusively neighborhood exposure
Lieben & Pistawka 1967 [14]	42 Pleural and peritoneal mesothelioma cases in southeast Pennsylvania	Insulation plant workers	3/42 (7%) cases had domestic exposure: 2 were daughters of insulation plant workers; 1 mother of two insulation plant workers	Amosite and chrysotile
Rusby 1968 [15]	Pleural mesothelioma in mother of factory workers	Asbestos factory workers	Mother of 3 daughters who worked in an asbestos factory	Laundered clothing for 1–2 years, 26 years prior; no other asbestos contact
Heller *et al.* 1970 [16]	10 Pleural mesothelioma cases at Massachusetts General Hospital 1960–1967	Pipefitter	1 woman (10%) washed her pipefitter husband’s dusty work clothes; husband had asbestosis	Clothes washing
Bittersohl and Ose 1971 [17] (as cited in NIOSH 1995 [7])	Wife of a chemical plant worker	Chemical plant worker	1 woman with pleural mesothelioma whose husband was exposed to asbestos insulation at a chemical plant	Clothes washing
Champion 1971 [18]	Son of lagger	Lagger	Patient was never occupationally exposed to asbestos; father was a lagger who wore work overalls home; emphysematous changes seen in mother; sister had pleural plaques.	---
Knappmann 1972 [19] (as cited in NIOSH 1995 [7])	Brother of asbestos worker	Asbestos factory worker	Case report of mesothelioma in a man who lived for several years with his sister who was an asbestos worker	---
Greenberg & Davies 1974 [20]	246 Pleural and peritoneal mesothelioma cases in England, Wales, Scotland (1967–1968)	Asbestos factory workers	2/246 (0.8%) with potential domestic exposures: 1 case had husband who worked in asbestos factory; 1 case lived near asbestos factory; parents worked at factory	Cases had 2 and 14 years of exposure, respectively
Lillington *et al.* 1974 [21]	Mesothelioma in husband and wife	Industrial exposure to asbestos	Husband had “industrial exposure”, wife washed his clothes; both were diagnosed with pleural mesothelioma	Clothes washing
Milne 1976 [22]	32 Pleural mesothelioma cases in Victoria, Australia	Asbestos cement factory	1/32 cases (3%) had domestic exposure; father worked in asbestos cement plant.	---
Edge & Chaudhury 1978 [23]	50 Mesothelioma cases from Barrow in Furness (British shipbuilding town; 1966–1976)	Shipyard plumber	1/50 (2%) was married to a shipyard plumber.	Crocidolite
Li *et al.* 1978 [24]	Family in which father was pipe insulator in a shipyard.	Shipyard insulator	Father had asbestosis and lung cancer; wife washed his clothes and had mesothelioma; daughter had mesothelioma.	Clothes washing
Epler *et al.* 1980 [25]	2 wives of asbestos workers	Asbestos factory workers	Mesothelioma in 2 wives of asbestos workers: 1 husband worked in an asbestos product factory for 23 years; 1 husband worked in an asbestos product factory and had asbestosis and mesothelioma.	---
Vianna *et al.* 1981 [26]	288 pleural and peritoneal mesothelioma cases in NY state (1973–1978)	Farmers, fireman	7/288 (2.4%) cases with potential indirect exposure (1 male, 6 females); 5 females lived with a farmer; 1 lived with a fireman.	---
Martensson *et al.* 1984 [27]	Two children of an asbestos worker	Foundry worker	Female with no occupational exposure; Father worked at foundry with insulation and hung his clothes where children played; Male, brother of female, grew up in same house and worked as a storekeeper for company supplying shipyard electrical equipment.	Exposure referred to as “slight household asbestos exposure during childhood”.
Krousel *et al.* 1986 [28]	Mother, daughter, and son with pleural mesothelioma	Factory workers	Mother worked as clothing sales person and candle-maker. First husband and second husband worked at lumber/shingle company. Family lived within a mile of lumber/shingle company that used asbestos wrap on pipes. Daughter worked as phone operator, husband was electrician. Son worked in submarine, shipyard, cement pipe maker, power company, and carpenter.	No microscopic evidence of asbestos fibers in son and daughter
Li *et al.* 1989 [29]	Family of asbestos worker	Insulator	Wife of insulator washed worker's laundry, used cloth sacks that were used to transport insulation as child's diapers. Child died of mesothelioma at age 32; mother died at age 49. Uncle who lived with family as teen and was briefly an insulator, developed mesothelioma at age 43. Father died of asbestosis at age 53.	Clothes washing and insulation cloth sacks as diapers.
Kane *et al.* 1990 [30]	10 Cases of mesothelioma in patients 40 years old and under	Asbestos factory worker, shipyard insulator	Of 10 cases, 5 had household exposure (50%): *Case 1:* Father delivered asbestos products; *Case 2:* Father worked at glass factory that made asbestos products; *Case 3:* Father worked as shipyard pipe insulator; lived 6 km from shipyard; mother had mesothelioma; father had adenocarcinoma; *Case 5:* Brother-in-law worked in asbestos plant; lived 2 km from asbestos factory; *Case 6:* Exposed to father's dusty work clothing for one year; older sister developed lung cancer with same exposure.	1–18 years of exposure
Konetzke *et al.* 1990 (German) [31]	48 Cases of mesothelioma from the National Cancer Register in East Germany and 19 cases of pleural plaques were investigated for non-occupational exposure to asbestos	---	22/48 (46%) cases caused by cleaning by members of the family of working clothes contaminated with asbestos.	Clothes washing
Oern *et al.* 1991 [32] (Norwegian; as cited in NIOSH 1995 [7])	Sister and husband of asbestos workers	Insulators	Family had 2 brothers, a sister and her husband. All males were insulators; 1 brother had asbestosis, other brother and sister had mesothelioma; woman who cleaned work clothes developed mesothelioma at age 79.	Clothes washing
Chellini *et al.* 1992 [33]	100 Cases of pleural mesothelioma in Tuscany, Italy (1970–1988)	Construction, plumber in chemical manufacturing	4/100 (4%) cases identified with “possible domestic” exposure—women whose husbands or members of the family were occupationally exposed (3 in construction and one as a plumber in chemical manufacturing) and who used to wash their spouses’ work clothes; same data also reported by Seniori-Constantini & Chellini 1997 [34].	Clothes washing
Dodoli *et al.* 1992 [35]	262 Cases of pleural mesothelioma in Leghorn and La Spezia, Italy (1958–1988)	Shipyard workers, oil refinery worker	10 (3.8%) women washed their relatives’ work clothes (9 shipyard workers, 1 oil refinery worker).	Clothes washing
Giarelli *et al.* 1992 [36]	170 Cases of mesothelioma in Trieste, Italy (1968–1987)	Shipyard workers	5/170 (2.9%) cases had domestic exposure and cleaned the clothes of their husbands who were shipyard workers.	80% had no AB **^a^**; 20% had few AB; Clothes washing.
Schneider *et al.* 1996 [37]	5 Pleural mesothelioma cases	Insulation mat manufacturing, turbine revision, roofer, asbestos cardboard manufacturing, and insulator.	“Causal relation established between the mesothelioma and inhalation of asbestos fibers while cleaning contaminated work-clothes and shoes”.	7–23 years of exposure; cleaning clothes and shoes
Seniori-Constantini & Chellini 1997 [34]	335 Pleural mesothelioma cases from registry in Tuscany, Italy (1970–1996)	NR **^b^**	30%–35% of 59 female cases were housewives; Same data source as Chellini *et al.* 1992 [33].	NR
Rees *et al.* 1999 [38]	123 Cases in South Africa	Mining workers	13/123 cases (11%) noted contaminated clothing as source of exposure, along with working with asbestos or living in mining district. “No subject exclusively exposed to contaminated work clothes brought home”. Three cases were reported to have only exposure to asbestos from contaminated clothing.	Mostly crocidolite and amosite; Contaminated clothing
Ascoli *et al.* 2000 (Italian) [39]	One female mesothelioma case	NR	Domestic exposure, duration of 20 years in an industrial town with a large chemical plant	NR
Barbieri *et al.* 2001 (Italian) [40]	190 Cases of mesothelioma in Brescia, Italy diagnosed 1980–1999	Asbestos hauler	1/190 (0.5%) had domestic exposure; wife of asbestos hauler who washed his clothes	Clothes washing
Bianchi *et al.* 2001 [41]	557 Malignant mesotheliomas of the pleura diagnosed 1968–2000 in the Trieste-Monfalcone area, Italy	Mainly shipbuilding town	21/65 (32%) females and 0/492 males had histories of domestic exposure, cleaning clothes of an asbestos exposed worker; includes Giarelli *et al.* 1992 [36] cases.	35% of domestic cases analyzed (n = 20) had AB; Clothes washing.
Mangone *et al.* 2002 (Italian) [42]	323 Pleural and peritoneal mesothelioma cases in Emilia-Romagna, Italy (1996–2001)	NR	13/325 (4%) were domestically exposed	NR
Miller 2005 [43]	32 Pleural and peritoneal mesothelioma cases gathered from law firms (since 1990)	Shipyard workers, insulators, others.	15 wives, 11 daughters, 3 sons, 1 sister-in-law, 1 niece, 1 boarder; Occupations of workers included: 13 shipyard workers, 7 insulators, 12 others	NR
Bianchi *et al.* 2007 [44]	99 Cases in Trieste, Italy (2001–2006)	NR	5 cases (5%) identified as “home exposure”, where patients had washed asbestos-exposed husbands’ work clothes	Clothes washing

**^a^** AB = Asbestos bodies; **^b^** NR = Not reported.

**Table 2:** Cohort and case-control studies of mesothelioma in domestically exposed populations.

Author and Year	Study Design	Population Studied (dates of death/incidence)	Controls/Unexposed	Disease	Fiber Type	Occupation of Worker(s)	Results **^a^**
Newhouse & Thompson 1965 a, b [2,3]	Case-control	76 cases from London hospital (1956–1963)	76 “in patient” series (patient in medical and surgical wards of the hospital during early summer 1964) matched by sex and date of birth	PL, PE	Crocidolite, chrysotile, amosite	Spinners, engine room worker, boiler coverer, asbestos factory foreman, docker, railway carriage builder, asbestos factory worker	9 cases had relative who worked with asbestos (7 pleural, 2 peritoneal) *vs*. 36 cases with no occupational exposure. 1 of “in patient” series had relative who worked with asbestos *vs*. 67 with no occupational exposure. **Crude OR = 16.75 (95% CI = 2.13–744.78) ^b,c,d^**
Ashcroft & Heppleston 1970 [45]	Case-control	22 cases in Tyneside (British shipbuilding town)	46 hospital controls matched for age and sex, free of malignant disease	PL, PE	NR	Asbestos worker	One case was the widow of an asbestos worker who, for a period of 3 years, had come home with asbestos dust on his hair and shoes.
McEwen *et al*. 1971 [46]	Case-control	80 cases from Scotland (1950–1967)	2 sets of hospital controls with coronary artery disease or lung or gastric cancer, matched for age and sex	PL, PE	For one case: “Blue and white” asbestos	For one case: dock worker	“...only a few [cases] had shared a household with relatives who were known to have worked with asbestos. There was no statistical difference with regard to either household or spare-time exposure to asbestos between the three groups [cases, cancer controls, cardiovascular controls]. One individual case, however, was interesting. The husband of one of the female cases had worked regularly with asbestos, both blue and white, as a dock labourer, and quite frequently had come home with asbestos on his overalls. His wife (the case) had washed them at home”.
Rubino *et al*. 1972 [47]	Case-control	50 cases from Piedmont, Italy (1960–1970)	Patients with same sex, age, and at same institution	PL	NR	Asbestos industry	3/50 cases had “family exposure” 0/50 controls had “family exposure” Thoracotomy cases: 1 (wife was employed in asbestos industry, no occupational exposure)/18 cases (3 with occupational exposure) 0 (no domestic or occupational exposure)/18 controls (no domestic or occupational exposure) No thoracotomy cases **^e^**: 2 (domestic exposure, unclear if could have occupational exposure)/32 cases (3 with occupational exposure) 0/32 controls (1 with occupational exposure)
Vianna & Polan 1978 [4]	Case-control	52 female NY state residents 20+ year old (1967–1977)	52 controls matched for age sex, race, marital status, county, year of death, and from non-cancer death	PL, PE	NR	Shoemaker, brake lining worker, pipefitter, heat insulation worker, heat electric wire worker, elevator insulation worker	10 patients had husbands/fathers working in asbestos industry (9 pleural, 1 peritoneal), whereas their matched controls did not. 1 control had husband working in asbestos industry, whereas their matched case did not: RR = 10 (95% CI = 1.42–37.40) **^e^**. 8 patients after excluded own occupational exposure, whereas their matched controls did not. 1 control had husband working in asbestos industry, whereas their matched case did not (*p* < 0.02). **OR = 8/1 = 8 (95% CI = 1–63.9) ^b^**
McDonald & McDonald 1980 [5]	Case-control	490 fatalities in Canada (1960–1972) and USA (1972) ascertained through 7,400 pathologists	Matched controls with pulmonary metastases from non-pulmonary malignancy by sex, age, year of death, and hospital	PL, PE	Chrysotile (at least 3 cases) and some amosite	Chrysotile production, insulation, or factory work	2 males, 6 females exposed to dusty clothing of asbestos worker; none among matched controls; 2 controls were exposed, but the paired cases were not (*p* = 0.08). **OR = 4.0 (95% CI = 0.43–9.42)^ b,e^**; 5/8 cases were exposed during childhood; 3/8 cases (1 control) were exposed by clothing of a Quebec chrysotile production worker; 5/8 cases (1 control) were exposed by clothing of a insulation/factory worker.
Muscat & Wynder 1991 [48]	Case-control	124 cases entering NY City hospital between 1981–1990	267 controls with non-tobacco disease, matched for age, sex, hospital, race, month of interview	M	NR	Auto mechanic	1/16 women without occupational exposure reported domestic contact with asbestos (one husband was auto mechanic); No information on controls; 1/105 males reported domestic exposure during childhood.
Spirtas *et al*. 1994 [49]	Case-control	208 cases from Veterans Administration hospital files and Los Angeles county and New York state cancer registries (1975–1980)	533 controls from death certificates or VA benefit files died of other causes excluding cancer, respiratory disease, suicide, or violence	PL, PE	NR	Asked if “cohabitant ever exposed to asbestos”. Separately asked if cohabitant performed any of 9 activities: (1) brake lining work/repair; (2) furnace/boiler installation/repair; (3) building demolition; (4) plumbing/heating; (5) insulation; (6) shipbuilding yard/repair; (7) elevator installation/repair; (8) production of textiles; (9) production of paper products.	OR for cohabitant ever exposed to asbestos: **Men: 13.2 (95% CI = 3.4–54.7) (12 pleural)** **Women: 3.4 (95% CI = 0.3–61.3)** **Crude OR = 6 (95% CI = 2.55–13.8) ^b,e^** OR for cohabitant performed any of 9 activities: Men: 12.1 (95% CI = 4.6–33.3) (34 pleural, 4 peritoneal) Women: 1.4 (95% CI = 0.3–5.6)
Howel *et al*. 1997 [50]	Case-control	185 cases of mesothelioma from mesothelioma and cancer registries and post-mortem records in Yorkshire, England (1979–1991)	159 controls from necropsy records excluded if had mesothelioma, bronchial or ovarian cancer, or circumstances that made gathering information difficult, matched for age, sex, and year of death	PL, PE	Unknown although crocidolite and amosite identified at factory that provoked concern for the study	NR	ORs for para-occupational exposure: Excluding subjects with likely occupational exposure: Likely *vs*. possible and unlikely 5.6 (95% CI = 1.9–16.5); Likely and possible *vs*. unlikely 1.8 (95% CI = 0.87–3.6); Excluding those with likely or possible occupational exposure: Likely *vs*. possible and unlikely 61.7 (95% CI = 3.4–1104); **Likely and possible *vs*. unlikely 5.8 (95% CI = 1.7–19.2).**
Case *et al*. 2002 [51]	Case-control	10 female residents aged ≥50 years of Quebec mining regions identified through hospital records (1970–1989)	150 controls selected from previously interviewed sample matched on age and area	PL	Chrysotile with some tremolite contamination (Thetford mines)	Chrysotile miners	10 cases identified: 9 (90%) had lived with one or more asbestos workers (*vs*. 65% of controls); Never lived with asbestos worker OR = 1 Lived with 1 or 2 workers OR = 3.4 (95% CI = 0.4–30.8); Lived with 3 or more workers OR = 9.0 (95% CI = 0.9–87.4) **Crude OR = 4.92** **(95% CI = 0.65–219.54) ^b,e^**
Magnani *et al*. 2000 [52]	Case-control	53 mesothelioma cases in Italy, Spain, Switzerland without occupational exposure (1993–1997)	232 controls from general population and hospitals without occupational exposure	PL	NR	Asbestos industry	OR for those with domestic exposure **^f^** and without environmental exposure: 4.92 (95% CI = 1.78–13.6); Probability domestic exposure **^f^** (adjusted for environmental exposure): Never exposed OR = 1; Low probability OR = 2.05 (95% CI = 0.83–5.09); Middle or high probability OR = 4.81 (95% CI = 1.77–13.1); Unknown OR = 0.74 (95% CI = 0.22–2.53); Intensity domestic exposure **^f^** (adjusted for environmental exposure): Never exposed OR = 1; Low intensity OR = 2.01 (95% CI = 0.84–5.06); Middle intensity OR = 5.68 (95% CI = 1.39–23.3); **High intensity OR = 7.83 (95% CI = 1.69–36.2)**; Unknown OR = 0.75 (95% CI = 0.21–2.69)
Welch *et al*. 2005 [53]	Case-control	24 male cases treated at Washington Cancer Institute, Washington, DC (1989–2001)	24 patients with appendical cancer treated at Washington Cancer Institute 1990–2000, matched for age and sex	PE	NR	Same 9 activities specified in Spirtas *et al.* 1994 [49], except brake lining work is grouped with tire work.	8/24 (33%) cases cohabitated with persons involved in 9 specified “high-risk-for-asbestos-exposure processes” 2/24 (8%) controls cohabitated with persons involved in 9 processes **Crude OR = 5.5 (95% CI = 0.89–57.95) for co-habitating with one of the nine activities.^b,e^**
Maule *et al*. 2007 [54]	Case-control	103 cases from Casale Monferrato, Italy (1987–1993)	272 controls matched by birth date, sex, vital status, date of death	PL	Crocidolite and chrysotile	Asbestos cement (AC) workers	OR for relative with AC occupation, adjusted for age, sex, and AC occupation: 2.4 (95% CI = 1.2–4.8); **RR for relatives’ AC occupation accounting for age, sex, and domestic (home materials) exposure:** **Including distance to plant = 1.4 (95% CI = 0.7–2.9)** Not including distance to plant = 2.1 (95% CI = 1.0–4.5) Update to Magnani *et al*. 2001 [55].
Rake *et al*. 2009/Peto *et al*. 2009 [56,57]	Case-control	622 patients in England, Wales and Scotland born since 1925 identified through physician records, cancer research network, and hospital records	1,420 population controls matched for age and sex	M	Suggests that higher death rate in UK is due to amosite use.	NR	**OR living with a potentially exposed worker before 30 years of age: 2.0 (95% CI = 1.3–3.2);** Logistic regression results: OR living with a potentially exposed worker before 30 years of age (women): 2.3 (95% CI = 1.5–3.8) OR living with a potentially exposed worker before 30 years of age (men): 1.1 (95% CI = 0.9–1.4) OR living with a high-risk parentor sibling: 1.3 (95% CI = 1.0–1.6) OR living with a high-risk spouse: 2.1 (95% CI = 1.3–3.5) See also tables of Peto *et al*. 2009.
Anderson 1982 [58]	Cohort	2,218 household contacts of Unarco amosite factory workers first employed between 1941–1945; 663 deaths	State of New Jersey, age and sex-specific	M	Amosite	Amosite insulation factory workers	After 30+ years from onset of exposure, mesothelioma deaths in 3/170 (1.8%) deceased household contacts (2 female and 1 male, all children of workers) **^c^** Observed/expected for respiratory cancer was 1.25 for females and 1.7 for males. Same cohort as Joubert *et al*. 1991 [59], Anderson 1979 [60]; Anderson 1976 [61], Selikoff 1981 [62].
Ferrante *et al*. 2007 [63]	Cohort	Cohort of 1,780 wives of asbestos cement workers in Casale Monferrato, Italy (deaths from Registrar’s office, incidence from mesothelioma registry) Deaths: 1965–2003Incidence: 1990–2001	Used age and sex specific rates in Piedmont, Italy for reference	PL, PE	Crocidolite and chrysotile	Asbestos cement workers	Peritoneal cancer SMR = 2.51 (95% CI = 0.52–7.35) Pleural cancer SMR = 18.00 (95% CI = 11.14–27.52) **Pleural malignant mesothelioma** **SIR = 25.19 (95% CI = 12.57–45.07) ^d,e^** Update to Magnani *et al.* 1993 [64].
Reid *et al*. 2008 [65]	Cohort	Followed 2,552 women and girls who lived in Wittenoom (crocidolite mining town) from 1943 to 1992 and were not involved in mining/milling (1950–2004)	Western Australia female population from 1970–2004	PL (0 PE)	Crocidolite	Crocidolite miners	The risk of death from mesothelioma was increased, but not significantly, in residents known to have lived with (HR = 2.67, 95% CI = 0.77–9.21) **^e^** or **washed the clothes of an Australian Blue Asbestos Company asbestos worker (HR = 2.61, 95% CI = 0.85–7.99) ^d^**; Update to Hansen *et al*. 1993 [66].
Bourdès *et al.* 2000 [10]	Meta-analysis	Five studies: Yazicioglu *et al*. 1980 [67]; Newhouse & Thompson 1965 [3]; McDonald & McDonald 1980 [5]; Magnani *et al*. 1993 [64]; Howel *et al*. 1997 [50]	--	PL	Chrysotile (McDonald & McDonald 1980 [5]); the rest are amosite or mixed fibers	--	Meta RR: 8.1 (95% CI = 5.3–12) **^e^**

**^a^** = Results in bold indicate values used in the meta- analysis; **^b^** = Relative risk estimate and/or 95% CI was calculated because it was not provided in the study; **^c^** = Appears exposure classification done in a hierarchy, so domestic cases and controls may contain subjects with neighborhood exposure (which itself is statistically significant); **^d^** = Potential confounding by neighborhood exposure; **^e^** = Potential confounding by occupational exposure; **^f^** = Domestic exposure included exposures to asbestos-containing materials at home. Several with “high” domestic exposure included “crushed asbestos material in the courtyard”; PL = Pleural mesothelioma, PE = Peritoneal mesothelioma, M = Mesothelioma, OR = Odds ratio, RR = Relative risk, CI = Confidence interval, SMR = Standardized mortality ratio; SIR = Standardized incidence ratio, HR = Hazard ratio; NR = Not reported.

**Table 3:** Epidemiologic studies of interstitial and pleural abnormalities in domestically exposed populations.

Author and Year	Study Design	Population Studied	Comparison Group	Disease	Fiber Type	Occupation of Worker	Results
Navratil & Trippe 1972 [68]	Cohort	114 Blood relatives of asbestos workmen in Czechoslovakia	“General population” of district N with no known exposure	Pleural calcifications	Chrysotile	Asbestos product plant	4/114 (3.5%) of blood relatives had pleural calcifications. Observed/expected = 4/0.39
Anderson 1982 [58]	Cohort	679 Household contacts (no occupational asbestos exposure) of amosite factory workers in Paterson, NJ employed between 1941–1954	325 urban NJ residence controls matched by sex, age, and residential community without asbestos exposure	Small opacities (≥1/0) and pleural abnormalities (pleural thickening, pleural calcification, pleural plaques) (1971 ILO **^a^**)	Amosite	Amosite asbestos factory workers	Household resident during index worker employment period. Cases (N = 679): Small opacities = 114 (17%), Pleural abnormalities = 178 (26%), Both = 239 (35%) (*p* < 0.001 compared to Controls). Controls (N = 325): Small opacities = 10 (3%), Pleural abnormalities = 6 (2%), Both = 15 (5%) Found statistically significant relationship between duration of exposure and year of first exposure and pleural thickening, pleural calcification, and both together, but not small opacities alone.
Ferrante *et al.* 2007 [63]	Cohort	Cohort of 1,780 wives of asbestos cement workers in Casale Monferrato, Italy (deaths from Registrar’s office: 1965–2003)	Used age and sex specific rates in Piedmont, Italy for reference	Nonmalignant respiratory disease	Crocidolite and chrysotile	Asbestos cement workers	SMR^ b^ = 0.86 (0.47–1.45)
Kilburn *et al.* 1986 [69]	Cross-sectional	274 Wives, 79 sons, and 140 daughters of shipyard workers from Long Beach, CA who started work before 1961. Subjects volunteered and had no occupational exposure.	1,347 Members of Long Beach Census tract in 1975 and random sample of adult population of Michigan during 1978–1979 without occupational asbestos exposure	Asbestosis and pleural abnormalities (refer to all as asbestosis) (ILO 1980, ≥1/0, and/or presence of pleural thickening or plaques)	NR	Shipyard workers	Asbestosis prevalence: Wives: 11% Sons: 8% Daughters: 2% Comparison populations: Long Beach men: 3.7% Long Beach women: 0.6% Michigan men: 0.5% Michigan women: 0.0% Wives with abnormalities: Pleural only: 39% Parenchymal only: 52% Parenchymal and pleural: 10% 75% of wives with asbestosis had husbands with asbestosis.
Sider *et al.* 1987 [70]	Cross-sectional	93 wives > 40 years of age of current and retired insulators screened from January to March 1986 at Northwestern Memorial Hospital in Chicago with no occupational exposure	Wives without radiographic abnormalities	Parenchymal opacities and pleural changes according to ILO 1980	NR	Pipe covers and asbestos removers (insulation workers)	18/93 (19.4%) demonstrated pleural changes consistent with asbestos exposure: pleural plaques (N = 16, 88.9%), diaphragm plaques (N = 6, 27.8%), pleural calcification (N = 3, 16.6%), and diffuse pleural thickening (N = 1, 5.5%). No parenchymal opacities. Radiographs of the husbands were compared for 17 of the 18 wives with pleural abnormalities. 14 of the husbands (82%) demonstrated more severe pleural involvement than their wives and 6 had parenchymal abnormalities. The remaining 3 wives with more severe changes had at least one household contact in addition to her husband. Only year of initial exposure was statistically different from the comparison group.
Peipins *et al.* 2003 [71]	Cross-sectional	6,668 Participants ≥ 18 years of age who had lived, worked, or played in Libby, MT for at least 6 months before December 31, 1990	None	Pleural abnormality (any unilateral or bilateral pleuralcalcification on the diaphragm, chest wall, or other site or any unilateral or bilateralpleural thickening or plaque on the chest wall, diaphragm, or costophrenic angle site, consistent with asbestos-related pleural disease, using the PA view, the oblique views, or a combination of those views) and interstitial abnormality (ILO 1980, ≥1/0).	Libby amphibole (tremolite, actinolite, winchite, richterite)	Vermiculite miners	Lived with W.R. Grace workers (n = 1,376): Pleural abnormality N = 358 (26.0%); Interstitial abnormality N = 17 (1.2%); Does not exclude occupational or non-occupational exposure; Using logistic regression found having been ahousehold contact of a vermiculite miner associated with pleural abnormalities.

**^a^** ILO = International Labour Organization disease classification; **^b^** SMR = Standardized mortality ratio.

The case reports and case series include pleural and peritoneal mesothelioma in wives, children, mothers, and siblings of asbestos workers such as miners, asbestos factory workers, pipefitters, laggers/insulators, and shipyard workers. Unfortunately, these published case reports rarely identified the type of asbestos to which the case was exposed [1,14,23,38], with a few exceptions, all of which reported exposure to amphibole asbestos (amosite or crocidolite) (Table 1). None of the case reports provided information on the level of asbestos exposure experienced in each case, although a limited number of studies reported results of lung-burden analyses [28,36,41]. Two of these studies reported finding asbestos bodies in 20% to 35% of persons examined [36,41]. Several of the case reports specifically noted clothes washing as the source of exposure via the inhalation pathway [15,16,17,21,24,29,31,32,33,34,35,36,37,38,40,41,44].

Among studies of the association between domestic exposure and asbestos-related disease, mesothelioma was the most common disease reported. Several cohort (n = 3) and case-control (n = 14) studies of mesothelioma evaluated domestically exposed populations or identified cases of mesothelioma in domestically exposed individuals (Table 2). One meta-analysis was also identified. The occupations of the workers included in the studies were primarily those associated with traditional high-risk trades: asbestos miners, asbestos factory workers, shipyard/dock workers, textile workers, furnace/engine/boiler room workers, railway carriage builders, pipefitters, and insulators. Our review included 14 case-control studies, of which 10 reported relative risk estimates or provided enough information to calculate a crude relative risk estimate [2,3,4,5,49,50,51,52,53,54,56], ranging from 1.4 [54] to 16.75 [2,3]. Two of the three cohort studies reported relative risk values [63,65]. In the first cohort study, a statistically significant standardized incidence ratio (SIR) of 25.19 (95% CI: 12.57–45.07) was reported for wives of Italian cement workers [63], although results were not adjusted for potential confounding by neighborhood or occupational exposure. In the second cohort study, a non-statistically significant hazard ratio (HR) of 2.61 (95% CI: 0.85–7.99) was reported in household members of workers of the Australian Blue Asbestos Company [65]. In this study, potential neighborhood exposures were also not evaluated in the estimation of relative risk. 

The cohort and case-control studies evaluated both pleural and peritoneal mesotheliomas, with some studies not discerning between the two sites. In many studies, asbestos fiber type was also not reported. The fiber type to which the study participants were exposed is an important factor, as amphibole fibers (crocidolite, amosite or tremolite) are generally more potent then chrysotile fibers [72,73,74,75]. When reported, the workers via whom the individuals were domestically exposed were nearly always exposed to amphiboles. This fiber-type issue is further complicated by the fact that some chrysotile deposits have different degrees of co-occurrence of tremolite. One case-control study evaluated exposure to chrysotile in 10 female co-habitants of Quebec chrysotile miners, although the miners worked in the Thetford area, which the authors described has having the highest tremolite content of the Canadian mining sites. This study resulted in a non-significant increase in the risk of mesothelioma (odds ratio [OR] = 4.92, 95% CI: 0.65–219.54) among co-habitants [51]. 

**Figure 1:**
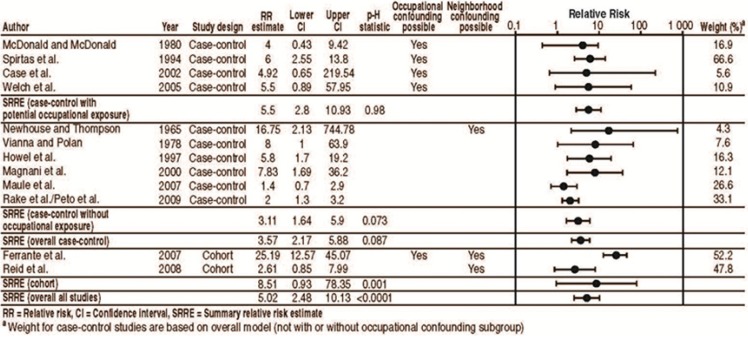
Meta-analysis of cohort and case-control studies of mesothelioma in domestically exposed populations.

Meta-analysis of all 12 cohort and case-control studies with reported relative risk estimates resulted in an SRRE of 5.02 (95% CI: 2.48–10.13; Figure 1). This SRRE indicates a statistically significant increase in the risk of mesothelioma for those domestically exposed, although heterogeneity was evident (p-H < 0.0001). The lower bound of the confidence interval in the Ferrante *et al.* study [63] is greater than the upper bound of the confidence interval from the overall summary effect. Removal of this study in a sensitivity analysis resulted in an attenuation, albeit still statistically significant, of the overall effect (SRRE = 3.34, 95% CI: 2.15–5.19), and the model became more homogeneous (p-H = 0.126).

A further sub-analysis by study type (cohort *vs.* case-control) was performed. The SRRE for the two cohort studies together [63,65] was elevated, but was not statistically significant (SRRE = 8.51, 95% CI: 0.93–78.35; p-H = 0.001). There is considerable heterogeneity between these two cohort studies; the disparity in risk estimates is likely due to potential confounding by occupational exposures (e.g., [63]) and neighborhood exposures (e.g., [63,65]). In contrast, the SRRE for the 10 case-control studies was elevated and statistically significant (SRRE = 3.57, 95% CI: 2.17–5.88; p-H = 0.087). Significant heterogeneity was present in both study design models. The case-control studies were further divided by whether the results could have been modified by the cases being occupationally exposed to asbestos themselves. The SRREs for the case-control studies, with and without potential modification by occupational exposure, were both statistically significantly increased, and the model of studies with potential occupational exposure was homogeneous (SRRE = 5.5, 95% CI: 2.8–10.93, p-H = 0.980 and SRRE = 3.11, 95% CI: 1.64–5.9, p-H = 0.073, respectively). Additionally, in the group of case-control studies without potential for occupational exposure, the highest relative risk value was from a study with increased likelihood of neighborhood exposure (16.75) [2,3]. As a sensitivity analysis, the cohort study by Reid *et al.* [65], which did not have potential occupational confounding, was analyzed with the six case-control studies that also did not have occupational confounding. The resulting SRRE is 2.87 (95% CI: 1.69–4.88). This value is not much different from the overall SRRE based on the six case-control studies alone, indicating that this study does not have a large effect on the analysis. As an additional sensitivity analysis, the case-control study by Newhouse and Thompson [2,3] was omitted from the six case-control studies that did not have potential occupational confounding. The relative risk estimate for this study is considerably greater than those for the other five. A point of deviation for Newhouse and Thompson [2,3] appears to be study date, which may be a proxy for increased exposure or for less accurate categorization of exposure compared to the more recent studies. As noted earlier, the Newhouse and Thompson studies included persons exposed to various fiber types, including chrysotile, amosite, and crocidolite. The resulting SRRE based on five case-control studies is 2.83 (95% CI: 1.51–5.31). This value is also not much different from the overall SRRE based on the six case-control studies, including Newhouse and Thompson [2,3], indicating that this study does not have a large effect on the analysis.

#### 3.1.2. Lung Cancer

The epidemiologic studies of domestic exposure rarely evaluated the risk of lung cancer. Only two studies with results for lung cancer were identified [58,63]. In the first study, a cohort of 2,218 family contacts of amosite asbestos factory workers in New Jersey first employed between 1941 and 1945 was studied [58]. The authors reported a slight statistically significant increase in cancer of the respiratory system for male family contacts of the factory workers with more than 20 years latency (observed *vs.* expected = 1.97), but not for female contacts (observed *vs.* expected = 1.70). In the second study of 1,780 wives of asbestos cement workers in Casale Monferrato, Italy, no significant increase in lung cancer was reported (SMR = 1.17; 95% CI: 0.60–2.04) [63]. Although the fiber potency gradient is less pronounced for lung cancer than it is for mesothelioma, fiber type is an important factor in determining disease. The study by Ferrante *et al.* included persons exposed to chrysotile and crocidolite, while the Andersen study included amosite workers.

#### 3.1.3. Pleural and Interstitial Abnormalities

Case reports of pleural and interstitial abnormalities in domestically exposed individuals date back to the 1960s [7,76,77,78,79,80] and focus primarily on pleural plaques. Epidemiologic studies of pleural (*i.e.*, plaques and diffuse pleural thickening) and interstitial abnormalities were gathered and reviewed (Table 3). As with the studies of asbestos-related malignancy, information on fiber type was either not reported or indicated a mixed fiber exposure. Six cohort and cross-sectional studies were identified [58,63,68,69,70,71], half of which accounted for potential confounding by occupational exposure [58,69,70,71]. Sider *et al.* [70] collected chest radiographs of the male workers and their wives, reporting that the majority (82%) of the husbands, who worked in the insulation trades, demonstrated more severe radiographic changes than their wives. Likewise, Kilburn *et al.* [69] reported that 75% of the wives with pleural and/or parenchymal abnormalities had husbands who worked in shipyards and exhibited abnormalities. One of these studies [58] also reported a statistically significant relationship between the duration of domestic exposure and year of first exposure with pleural thickening, calcification, or both abnormalities combined, but not small opacities alone. Sider *et al.* [70] reported that only the year of initial domestic exposure was statistically different from the comparison group. 

### 3.2. Review of Domestic Exposure Studies

Unfortunately, none of the epidemiologic studies reported the level of asbestos exposures experienced by the domestic cases themselves. This was expected, given the findings of previous review articles, and the difficulty of characterizing exposures in a domestic setting in an epidemiologic study. At best, the epidemiologic studies characterized exposure by intensity (low, medium, high) or probability of exposure. In our review of household exposure studies, nineteen separate exposure studies were identified, although some reported on overlapping populations. These studies, in each of the four categories of interest, are shown in Table 4. As with the epidemiology studies, most exposures were to mixed fibers. 

#### 3.2.1. Exposures in the Home Environment

Three of the studies reviewed provided results of sampling within the homes of asbestos workers [81,82,83]. Two of the three studies [81,83] reported airborne asbestos concentrations, while the third [82] summarized reports of fibers found in the settled dust. These three studies were primarily reviews or articles that reported exposure concentrations indirectly and did not provide sufficient information to attribute concentrations directly to worker clothing. For example, in their book, Selikoff and Lee [82] described a study performed by Mount Sinai regarding asbestos workers’ homes, wherein workers were employed at asbestos factories during 1941 to 1954, and “small amounts” of amosite were identified in settled dust in the workers’ homes and in neighboring homes of non-asbestos workers up to 400 yards downwind of factories. The authors attributed these amosite fibers found in workers’ homes to the clothing workers brought home from the workplace. The amosite fibers identified in the homes of non-asbestos workers were attributed to atmospheric contamination and deposition; however, because samples were collected 20 to 25 years after the fact, it is difficult to attribute concentrations directly to a take-home source such as clothing. In addition, these samples involved settled dust from surfaces in the homes, rather than airborne asbestos concentrations. The observed dust concentrations are not representative of the air inhaled by household members. 

In 1986, the World Health Organization (WHO) reported a mean concentration of 0.006 f/cc (range, 0.002–0.011 f/cc) in the homes of South African asbestos miners and estimated a range of 0.01–1 f/cc for “paraoccupational” exposures [83]. Although described as an environmental study, Nicholson *et al.* [81] found levels ranging from 100 to up to 5,000 ng/m^3^ by weight (approximately 0.003–0.15 f/cc based on the conversion factor presented by the National Research Council [84]) in the homes of chrysotile miners in California and Newfoundland, where homes were described as having visible fibers and dust in living areas and laundry facilities.

#### 3.2.2. Exposures from Clothing

Our literature review identified only one study that provided airborne asbestos levels measured during laundering of workers’ clothing [85]. This study evaluated concentrations associated with laundering clothes contaminated during an asbestos removal operation, reporting an average airborne concentration of 0.4 ± 0.1 f/cc (duration not specified) resulting from picking up contaminated clothing and loading it into the washer. No information was provided regarding specific sample duration; however, earlier evaluations performed at the same building reported mean fiber counts that were typically associated with one-hour sampling duration. The exposure levels “dropped to zero” following a single wash cycle (Table 4). A maximum personal sample of 1.2 f/cc (corresponding mean = 0.4 f/cc, sample duration unknown) was measured during the complete laundry operation, and all asbestos fibers detected were chrysotile. This study was not conducted in a home laundry setting, but focused primarily on the sufficiency of the decontamination procedures used by 40 workers after the removal of an asbestos-containing ceiling. Although not reported specifically as 8-hour time-weighted averages, these exposure levels are clearly low.

Two studies regarding bulk samples of dust on workers’ clothing performed by NIOSH at friction product manufacturing plants were also reviewed. Unfortunately, these studies did not discuss airborne exposures resulting from this dust [86,87]. One of these studies reported that asbestos was present in 85% of samples obtained from clothing and car seats of friction workers, but did not describe the fiber type.

**Table 4:** Domestic exposure studies.

Author and Year	Population or Task Studied	Asbestos Fiber Type	Reported Exposure Information	
*Studies reporting measurements of airborne or settled dust in homes of asbestos workers*				
Nicholson *et al.* 1980 [81]	Homes of chrysotile miners in Copperopolis, California and Baie Verte, Newfoundland	Chrysotile		Homes of miners: 100 to < 5,000 ng/m^3^ (approx. 0.003–0.15 f/cc **^a^^,b^**) (n**^c^** = 13) Homes of non-miners (Baie Verte): 32, 45, 65 ng/m^3^
Selikoff and Lee 1978 [82]	Settled dust in asbestos workers’ homes	Amosite		“...small amounts of amosite were found 20–25 years later in the settled dust of asbestos workers' houses from factory operations over the period 1941–1954, and up to 400 yards downwind in the neighboring houses of nonasbestos workers”.
WHO **^d^** 1986 [83]	Asbestos miners’ homes	NR		Residences of asbestos miners in South Africa: Mean = 0.006 f/cc (range, 0.002–0.011 f/cc); Para-occupational range: 0.01–1.0 f/cc
*Study of clothing and laundering*				
Sawyer *et al.* 1977 [85]	Asbestos abatement workers	Chrysotile		Mean of personal samples (n = 12): 0.4 f/cc (max = 1.2 f/cc) Mean of area samples: During picking up clothing (n = 4): 0.4 f/cc Loading washer (n = 5): 0.4 f/cc Loading dryer (n = 6): 0.0 f/cc
*Exposure simulation studies*				
Jiang *et al.* 2008 [88]	Unpacking and repacking clutches	Chrysotile		30 min PCM^ e^ -adjusted mean following clothing handling = 0.002 ± 0.002 f/cc (n = 4) Estimated 8 h TWA^ f^= 0.0001 f/cc.
Madl *et al.* 2008 [89]	Unpacking and repacking brakes	Chrysotile		30 min PCM-adjusted mean (range) following clothing handling (n = 5): 0.011 f/cc (0.002–0.015 f/cc )
Madl *et al.* 2009 [90]	Mechanics performing brake repair on heavy equipment	Chrysotile		30 min PCM-adjusted mean (range) following clothing handling: For primary worker (n = 2): 0.036 f/cc (0.032–0.039 f/cc) For bystander (n = 2): 0.010 f/cc (0.003–0.018 f/cc)
Mowat *et al.* 2007 [91]	Roofers removing dried material from laundered clothing	Chrysotile		30 min PCM-E^ g^ mean (n = 12): 0.0017 f/cc (range = 0–0.011 f/cc) Calculated TWAs = 0.003–0.002 f/cc
Weir *et al.* 2001 [92]	Brake mechanics	Chrysotile		Agitation of operator’s coveralls (30 min) = 0.72 f/cc Background concentration in laboratory ≤ 0.065 f/cc
*Lung burden studies*				
Dawson *et al.* 1993 [93]	Women with mesothelioma (n = 170)	Mixed		Women with domestic exposure (n = 14): Total amphiboles = 4.9 × 10^6^ f/g **^h^** (range = 0–251) Chrysotile = 12.7 × 10^6^ f/g (range = 0–2506) Control group (n = 31): Total amphiboles = 0.04 × 10^6^ f/g (range = 0–1.0); Unknown = 4.4 × 10^6^ f/g (range = 0–20.1)
Dodson *et al.* 2003 [94]	Women with mesothelioma (n = 15)	Mixed		4 women had potential domestic exposure through their father’s/husband’s work; 2/4 had ferruginous bodies (wife of crocidolite worker and wife of laborer/ship scaler/cement worker/*etc*.); 1 had uncoated amosite and tremolite (daughter of shipyard worker); 1 had uncoated tremolite and no commercial amphiboles (daughter of maintenance worker and wife of shipyard worker/painter/*etc*.
Giarelli *et al.* 1992 [36]	Family members of shipyard workers with mesothelioma in Trieste, Italy	---		5/170 (2.9%) cases had domestic exposure, cleaned clothes of spouse:80% had no AB **^i^** 20% had few AB (1–5 AB/section)
Gibbs *et al.* 1989 [95]	Mesothelioma cases with para-occupational exposure (n = 13)	Mixed		Mean (range) fiber counts of Group II para-occupational, e.g., wives of males working with asbestos (n = 13): Total: 277.8 (5.6–2507) Amosite: 1.5 (0–6.1) Crocidolite: 31.8 (0–251.1) Chrysotile: 218.9 (1.9–2507) Mean (range) fiber counts of Group V, unexposed (n = 21): Total: 42.5 (0–188.3) Amosite: 0.7 (0–4.6) Crocidolite: 5.5 (0–101.7) Chrysotile: 19.6 (0–76.5) Units in fiber × 10^6^/g dry lung
Gibbs *et al.* 1989 [95]	Mesothelioma cases without occupational exposure (n = 84).	Mixed		Para-occupational group averages (range) in dry lung: Amosite: 1.5 × 10^6^ f/g (0–6.1) Crocidolite: 31.8 × 10^6^ f/g (0–251) Chrysotile: 218.9 × 10^6^ f/g (1.9–2507) Unexposed group averages (range) in dry lung: Amosite: 0.7 × 10^6^ f/g (0–4.6) Crocidolite: 5.5 × 10^6^ f/g (0–102) Chrysotile: 19.6 × 10^6^ f/g (0–77)
Gibbs *et al.* 1990 [96]	Mesothelioma cases with para-occupational exposure (n = 10)	Mixed		9 exposed to their husbands’ work clothes and 1 was the daughter of a man who had died of asbestosis.
Huncharek 1989 [97]	Wife of shipyard machinist	Mixed		Chrysotile: 2.5 × 10^6^ f/g ACj: 0.8 × 10^6^ f/g TAA**^k^**: 3.2 × 10^6^ f/g (in dry lung)
Roggli & Longo 1991 [98]	Women whose only known exposure was household contact with an asbestos worker with asbestos-related disease (n = 6)	NR		Household contacts: median = 1,700 AB/g (range, 2–8,200) Uncoated fibers (UF **^l^**): median = 24,300 UF/g (range, 17,000–120,000) Normal range: median = 3,100 UF/g (range, 0–20)
Roggli 1992 [99]	Household contacts with mesothelioma (n = 3)	NR		Wife of shipyard insulator: 8,200 AB/g (29 yr exposure) Daughter of insulator: 2,330 AB/g, 17,000 UF/g (25 yr exposure) Wife of shipyard worker: 2 AB/g, 24,300 UF/g (1–2 yr exposure) Normal lungs: 0–22 AB/g, 1,600–5,600 UF/g
Roggli *et al.* 2002 [100]	Household contacts with mesothelioma (asbestosis confirmed in 8.3%)	Mixed		Mean (range) lung burden in wet lung of household: 130 AB/g (2–14,100) AC: 3,400 f/g (450–116,000) TAA: 5,200 f/g (980–22,400) chrysotile: 1,800 f/g Mean (range) lung burden in wet lung of reference cases: AB: 3 f/g (2–22) AC: <600 f/g (<100–<2,540) TAA: 158,000 f/g (1700–455,000) Chrysotile: <600 f/g (<100–<2,540)

**^a^** based on conversion factor in NRC 1984; **^b^** f/cc = fibers per cubic centimeter; **^c^** n = number of samples or cases; **^d^** WHO = World Health Organization; **^e^** PCM = Phase contrast microscopy; **^f^** TWA = time-weighted average; **^g ^**PCM-E = phase contrast microscopy equivalents; **^h^** f/g = fibers per gram lung; **^i^** AB = Asbestos bodies; **^j^** AC = commercial amphiboles (amosite + crocidolite); **^k^** TAA = noncommercial amphiboles (tremolite + actinolite + anthophyllite) **^l^** UF = uncoated fiber;

#### 3.2.3. Exposure Modeling and Simulation

Five exposure simulation studies were identified (Table 4). Four of these involved an evaluation of simulated domestic exposures resulting from those working with friction products, such as brakes and clutches [88,89,90,92], three of which were performed by the same group of investigators. The fifth study characterized exposures from roofers’ clothing [91]. Phase contrast microscopy (PCM) was used in all simulations; transmission electron microscopy (TEM) was also used in all except the Weir *et al.* study [92] to analyze fiber type in clothing-related samples. In studies employing TEM, PCM-equivalent (PCM-E) concentrations were also reported.

The simulations of friction-product-related exposures involved laundering activities by agitating a brake mechanic’s coveralls [92] and simulated clean-up of countertops and clothes-handling tasks, such as shaking and folding clothes worn by an operator, whose work activities involved packing and re-packing boxes of brakes and clutches [88,89] or performing repair work on heavy equipment [90]. All of these studies involved exposures only to chrysotile asbestos of unknown origin, because this was the fiber type used in the formulation of asbestos-containing friction materials [101]. Estimated 30 min PCM-E mean values were reported as 0.002 ± 0.002 f/cc (8 h time-weighted average [TWA] = 0.0001 f/cc) and 0.002–0.015 f/cc (mean = 0.011 f/cc) during clothes handling following unpacking and re-packing of clutches and brakes, respectively [88,89]. Similar asbestos levels were reported by Mowat *et al.* [91] in the simulation of potential exposures from asphalt-based roofing materials from scraping or picking dried material from laundered coveralls, with a 30 min exposure value of 0.0017 f/cc (range, non-detect [ND]–0.011 f/cc). For mechanics performing brake repair on heavy equipment, equivalent 30 min mean values following clothes handling were 0.036 f/cc and 0.010 f/cc for primary workers and bystanders, respectively [90]. During agitation of a brake mechanic’s coveralls following brake work, the 30 min concentration was 0.72 f/cc [92]. 

#### 3.2.4. Lung-Burden Studies

**Figure 2:**
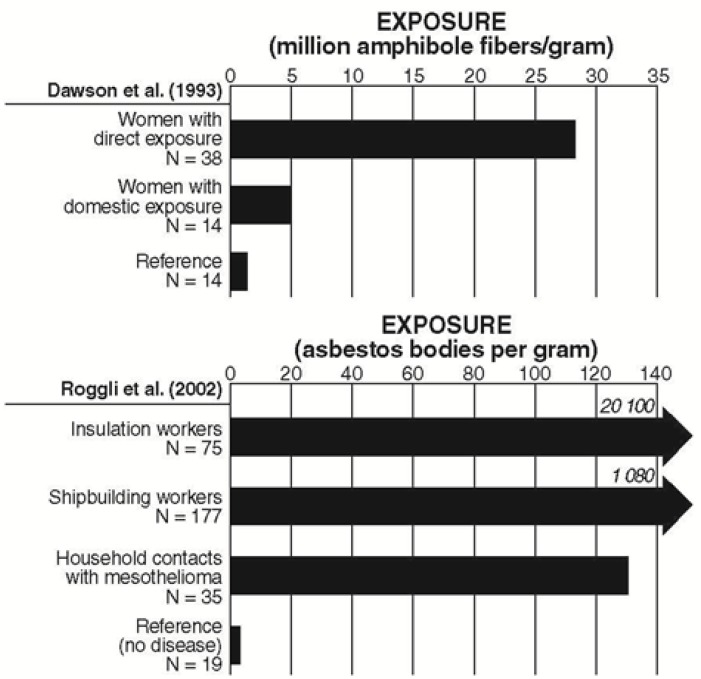
Lung-burden studies.

Six unique lung-burden studies were identified that provide results related to domestic exposure, generally reporting fiber concentrations either as fibers × 10^6^/g dry lung (f/g) or asbestos bodies per gram of lung tissue analyzed (AB/g). Gibbs and colleagues [95,96] and Roggli and colleagues [102] reported multiple times on overlapping populations. Asbestos bodies are indicative of amphibole exposures, because asbestos bodies form primarily on amphibole fibers [102]. Of the studies identified, most reported that the domestically exposed persons were typically wives or daughters of insulators, boilermakers, or shipyard workers [36,93,94,96,97,98,99,100]. All six studies identified the fiber type detected in the lung tissue examined and found amphibole asbestos fibers, such as crocidolite and amosite, in the lungs of domestically exposed persons (Table 4). Only two studies [93,100] presented lung-burden data for domestic contacts compared to a reference group (Figure 2). Both studies indicated significantly higher concentrations of amphibole asbestos and/or AB/g of lung tissue in domestically exposed cases compared to the reference group, and even higher concentrations of amphibole asbestos in directly exposed insulation or shipyard workers, although the domestically exposed persons and directly exposed workers were not linked. No study compared the lung burdens of workers with those of their spouses.

In the series of studies by Roggli and colleagues, all of the asbestos workers were diagnosed with asbestosis, and three with lung cancer; all of the household contacts were diagnosed with either mesothelioma or lung cancer. In an update to their analyses involving 1,445 cases of mesothelioma, Roggli and colleagues reported that, in the household contacts identified, 57% were found to have pleural plaques, and 7.9% had asbestosis [100]. Of the four domestically exposed cases evaluated in their study, Dodson *et al.* [94] found ferruginous bodies in lung tissue of two of the four women, uncoated commercial amphibole asbestos fibers in another woman, uncoated non-commercial amphibole asbestos fibers in a third woman, and chrysotile fibers in another. Not surprisingly, high concentrations of crocidolite fibers were identified in lung tissue of the spouse of the crocidolite cement worker.

Some of the studies provided exposure estimates of those domestically exposed, but generally, the objectives for these studies were not related to an evaluation of domestic exposure, and no explanation of exposure-level estimation or quantitative analysis was performed. For example, Camus *et al.* [103] analyzed lung cancer risk among women living in asbestos mining areas wherein indoor household concentrations were estimated by extrapolation from fiber burden results in ten autopsied women who had lived with asbestos workers. Indoor asbestos concentrations associated with these observed fiber burdens were reported as being approximately 0.03 f/cc higher than existing outdoor levels, although the method by which this result was obtained was not described. These authors also reported an estimated cumulative exposure of 7.8 f/cc-years in household contacts using their approach.

## 4. Discussion and Conclusions

Overall, the results indicate a consistent elevated risk of mesothelioma in the domestically exposed populations, and summary results suggest that the association may be modified by the potential for additional occupational exposure. The SRRE for all cohort and case-control studies indicated a five-fold greater risk of mesothelioma for persons domestically exposed. For persons domestically exposed, the results of the meta-analysis indicated a three- to five-fold increased risk for case-control studies and 8.5-fold risk of mesothelioma for cohort studies, although the cohort studies suffered from heterogeneity (and there were only two studies). Comparatively, the Bourdès *et al.* [10] meta-analysis of pleural mesothelioma found an eight-fold greater risk. Our finding of increased risk applies to domestically exposed populations in which the associated workers were employed in traditionally high-risk occupations involving exposure to asbestos, where in many cases, possible confounding due to direct asbestos exposures was not taken into account. For most of the included studies, exposures were to amphibole or mixed fiber exposures associated with traditionally high-risk occupations.

The domestic exposure studies of lung cancer were extremely limited and not supportive of an association between domestic exposure and lung cancer. In addition, both identified studies suffered from potential confounding by other occupational exposures and lack of consideration of smoking history. Fiber type was also not considered in these studies, though the two identified studies specifically included those exposed to amphibole asbestos. For the studies of pleural and interstitial abnormalities, results of pleural and interstitial abnormalities were often combined, despite them being two separate and distinct disease types, with reported exposures primarily being to mixed fibers. Even within pleural abnormalities themselves, the disease types differ (*i.e.*, pleural plaques *vs.* diffuse pleural thickening) in terms of their health impact and level of exposure required to cause the abnormality [104]. The studies supported an association between abnormalities and domestic exposure, but the association is largely due to pleural abnormalities. Similar to the mesothelioma studies, the workers themselves were likely highly exposed populations with exposure to amphiboles (e.g., asbestos product plant, amosite factory, and shipyard workers, insulators, and miners). These studies are unique, in that they provide linked data on husbands and wives (*i.e.*, data were collected on husbands and their wives, rather than workers in general and wives in general).

The findings of the lung-burden studies are consistent with the epidemiologic studies, in that they concluded that accumulated fiber burdens in persons exposed domestically might suggest a significant risk of mesothelioma, although the directly exposed workers in these studies were in traditional high-risk occupations, such as insulators, shipyard workers, and those in the building trades. All nine lung-burden studies (six unique studies total) detected amphibole fibers in the lungs of domestically exposed persons and, when compared to an appropriate reference group, were found to be present at significantly higher concentrations (Figure 2). In the Roggli series of studies, the lungs of household contacts were found to contain commercial amphiboles (defined as amosite and crocidolite) in 48% of cases, non-commercial amphiboles in 10.5% of cases, and chrysotile in 4.2% of the cases. Other studies also reported elevated amphibole fiber burdens [93,95,97]. These concentrations were reported as similar to those found in construction workers (190 AB/g), with higher lung fiber burdens reported in wives than children of these workers [100].

Ideally, airborne exposure estimates including asbestos fiber type information for the participants in the epidemiologic studies would exist in the peer-reviewed literature to allow for better evaluation of risk; however, this is not the case for epidemiologic studies of domestically exposed individuals. Instead, there are review articles with limited discussion of airborne measurements in asbestos miners’ homes, one study of airborne monitoring during laundering the clothes of asbestos abatement workers exposed to chrysotile, and more recent controlled simulation studies of airborne concentrations during the handling of the clothes of workers who traditionally have low chrysotile exposures. Thus, the experiences of the domestically exposed populations in the epidemiologic studies (exposed via workers in high-risk occupations, with high levels of exposure to amphibole asbestos) do not correspond to the exposures characterized by the available airborne data (generally for low-level chrysotile exposures).

As noted above, the existing relevant airborne exposure data pertain to populations occupationally exposed to low-level chrysotile asbestos. Given the absence of epidemiologic studies of populations exposed domestically by family members who were exposed occupationally to low-level chrysotile, alternative methods must be used to estimate the exposures and risk of mesothelioma for these populations. 

First, it is logical that, if the worker is exposed to low levels of asbestos occupationally, then their co-habitants would experience even lower exposure concentrations. Automobile mechanics are a good population in which to test this hypothesis, because brake mechanics are exposed to low concentrations of solely chrysotile asbestos (e.g., [105,106]). For example, Paustenbach *et al.* [105] reported a typical 8-hour TWA exposure of 0.04 f/cc for automobile mechanics, based on review of numerous historical studies. When 8 h TWAs are calculated for the four simulation studies that involve clothing manipulation or potential take-home exposure from friction products [89,90,92], the exposure levels reported are approximately two orders of magnitude lower than the 8-hour TWA for automobile mechanics (0.0001 f/cc *vs.* 0.04 f/cc). In fact, the daily exposures resulting from clothing activities were indistinguishable from background concentrations of asbestos, reported as ranging between 0.00001 f/cc and 0.0001 f/cc [107].

The results of the simulation studies are based on a small sample size in some studies (n = 1 in Jiang *et al.* [88]) or involved a short period of time (45 seconds in Jiang *et al.* [88], to 2 min in Madl *et al.* [89]); however, in all four studies, the results were consistently low, well below current and historical occupational exposure limits and, in some cases, within ambient concentrations. The anomaly of higher concentrations reported in the Weir *et al.* [92] study can be explained, because the majority of the fibers present in the sample were non-asbestiform, such as cotton fibers. Although this comparison has limitations due to the small sample sizes and exposure durations attributed to clothing manipulation activities and differences in methods used to analyze for asbestos fibers, the comparison nonetheless indicates that, at a minimum, domestic asbestos exposures to persons derived from domestic relationships with automobile mechanics are likely to be lower than those observed in occupationally exposed career automobile mechanics. This is consistent with the lung-burden studies showing a gradation of fiber burden from occupationally exposed to domestically exposed persons [93,100].

In our review, only one study [48] identified a domestically exposed case of mesothelioma reportedly due to chrysotile exposure in a woman whose husband was an automobile mechanic. Although fiber type was not specifically reported, chrysotile was the only fiber type used in the manufacture of brake and clutch parts [101]. This study, and therefore this case, was not included in the meta-analysis, because it lacked the information to calculate an estimate of relative risk, namely a comparison group. Vianna and Polan [4], Spirtas *et al.* [49], and Welch *et al.* [53] combine the activity of brake lining work/repair with traditionally highly exposed asbestos activities (e.g., insulation, shipyard work); thus, any observed increase in risk cannot be attributed to automobile mechanic work or solely to chrysotile exposure, and instead is highly likely attributable to the other activities (e.g., [108]). 

Second, if workers whose occupation involving low-level chrysotile exposure is not associated with an increased risk of mesothelioma, it follows that co-habitants of these workers also would not have an increased risk of mesothelioma. The existing epidemiologic studies of domestically exposed populations support this hypothesis, and demonstrate that the risk for the domestically exposed individual is remarkably less than that of the worker. While the exposure data is not complete in many of these studies with respect to both exposure level and fiber type, at least for one group—mechanics—the epidemiology shows that career workers exposed to low levels of chrysotile asbestos are not at risk and, therefore, it follows that the families of these workers would also not be at increased risk for developing asbestos-related disease. This has also been demonstrated in other industries, where higher exposures have been reported. Maule *et al.* [54] provided risk estimates for those occupationally exposed during asbestos cement manufacturing, and their relatives, with the OR for the workers being remarkably greater than for those domestically exposed (27.5 *vs.* 1.4, non-significant). Likewise, the radiographic studies showed that the majority of the workers demonstrated more severe radiographic changes than their wives, and alternatively, if the wives showed radiographic abnormalities, so did their husbands [69,70]. Thus, if the existing studies of domestically exposed populations show trends of lower risk and disease than the worker population, it follows that if the worker population does not have increased risk, then the domestically exposed co-habitant would not either. 

In conclusion, the epidemiologic and lung burden studies, as a surrogate of past exposure, support an increased risk of mesothelioma and interstitial, but more likely pleural, abnormalities in domestically exposed individuals whose associated worker was employed in traditionally high-risk occupations involving exposure to amphibole asbestos. Quantifiable exposure concentrations do not exist for these domestically exposed cohorts; however, some data exist for manipulation of worker clothing after low-level chrysotile exposure, mostly in the form of recent exposure simulations. These simulation data show that results for domestic exposures are lower than the workers’ exposures and are commensurate with background concentrations.
